# Development of a transition readiness score for adolescents living with perinatally-acquired HIV and transitioning to adult care

**DOI:** 10.1007/s10461-022-03650-4

**Published:** 2022-04-01

**Authors:** Brian C. Zanoni, Nicholas Musinguzi, Moherndran Archary, Thobekile Sibaya, Jessica E. Haberer

**Affiliations:** 1 Emory University, Atlanta, Georgia United States of America; 2 Children’s Healthcare of Atlanta, 2015 Upper Gate Drive NE, 30322 Atlanta, Georgia United States of America; 3 University of KwaZulu-Natal Nelson Mandela School of Medicine, Durban, South Africa; 4 King Edward VIII Hospital, Durban, South Africa; 5 Global Health Collaborative, Mbarara, Uganda; 6 Massachusetts General Hospital, Boston, Massachusetts United States of America; 7 Harvard Medical School, Boston, Massachusetts United States of America

**Keywords:** Healthcare Transition, Perinatally-acquired HIV, Transition readiness, Adolescents

## Abstract

We created a transition readiness score for adolescents with perinatally-acquired HIV as they transition from pediatric to adult care. Of the 199 adolescents who transitioned to adult care, 84 (42%) had viral suppression (< 200 copies/ml) one year after transition. Adolescents on first-line ART, with documented HIV status disclosure, and higher rating on the HIV Adolescent Readiness to Transition Scale had significantly higher odds of viral suppression after transition. Conversely, females, those with older age at ART initiation, and those with prior alcohol use had significantly lower odds of viral suppression after transition. Using these data, we created a transition readiness score organized into low, intermediate, and high levels of transition readiness. This transition readiness score can be used to identify adolescents who are likely ready to transition to adult care and identify additional areas for intervention to improve the likelihood of successful transition for those with lower transition readiness scores.

## Introduction

South Africa has an estimated 330,000 adolescents living with perinatally-acquired HIV, the highest of any country in the world (1, 2). As these adolescents age into adulthood, many of them require transition from pediatric- to adult-based clinical care for ongoing managment. However, the optimal timing and preparation for this healthcare transition are not known, nor are factors associated with successful transition. In addition, clinical guidelines on transition care lack evidence-based decision tools to assist clinicians in determining the timing of transition, often resulting in aged-based transition instead of assessing an individual adolescent’s transition readiness (3–6). The lack of individualized transition readiness assessments, reliance on aged based-transition and unclear focus for transition preparation likely contributes to the high rates of loss to follow-up, virologic failure, and death among adolescents living with perinatally acquired HIV after transiting to adult care (7–10).

In South Africa, retention in care among adolescents living with HIV who transition to adult care is suboptimal (11). This transition to adult HIV care may require that adolescents change institutions. As many as one-third of South African children and adolescents living with HIV never have a recorded visit at their transfer clinic and an additional 20% are lost after initial successful transfer (12). When transition occurs within the same institution, another study found retention among adolescents who transitioned to adult care to be lower than adolescents remaining in pediatric care (10). Moreover, the continuum of care for adolescents living with HIV in South Africa suggests that less than 50% are virally suppressed, even before the transition to adult care (9, 13).

In other settings, transition readiness assessments are used for adolescents with chronic medical conditions such as sickle cell disease, cystic fibrosis, childhood cancer, and chronic renal disease to assist with timing of transition to adult care (14–16). These transition assessments have not been validated based on clinical outcomes limiting their usability in clinical practice. In addition, they do not account for the complex psychosocial aspects of HIV care, such as HIV stigma, HIV status disclosure, or variable health literacy. Furthermore, they were developed in North America, which has important cultural and logistical differences with sub-Saharan Africa where the vast majority of adolescents with HIV live. A practical transition readiness assessment is needed to assist clinicians in determining what factors should be evaluated prior to transition to adult care. A transition readiness assessment can assist clinicians in determining which adolescents are ready to transition, when should they transition to adult care, and what preparation can be done for those not yet ready to transition.

To address this gap in adolescent HIV care, we identified factors associated with successful transition from pediatric to adult care for adolescents living with HIV in South Africa. We then used these factors to create a transition readiness score to assist clinicians in determining readiness and identifying modifiable factors to prepare for transition.

## Methods

### Setting

Prince Mshiyeni Hospital is a regional/district hospital located in the township of Umlazi outside of Durban, South Africa. The outpatient pediatric HIV clinic provides treatment for > 800 children and adolescents living with HIV— all of whom will require transition to adult care. In this setting, transition to adult care typically occurs after an adolescent’s 12th birthday but may occur later based on clinician judgement. Adolescents initially transition to the adult clinic at Prince Mshiyeni Hospital before being transferred to local clinics at a later date.

### Participant selection and enrollment

We recruited study participants from the pediatric clinic of Prince Mshiyeni Hospital during their routine clinic visits between August 2017 and May 2019. We offered enrollment to a convenience sample of consecutive adolescents (age > 12 years old) living with perinatally-acquired HIV who were transitioning from pediatric to adult care. To avoid accidental disclosure of HIV status by participating in the study, adolescents were referred to the research team by their primary pediatrician after they felt that the adolescent sufficiently understood their HIV status. To assess transition readiness at the point of transition, we enrolled participants on their last day in the pediatric clinic prior to transitioning to the adult clinic. In this exploratory study, we anticipated that a sample size of 200 participants and viral suppression rates of approximately 50% after transition would give sufficient power to evaluate up to ten independent variables using the heuristic rule of ten events for every independent variable. Adolescents with developmental delay interfering with the consent procedures were excluded from participation. Adolescents < 18 years old assented to study participation and written consent was obtained from the primary caregiver. Adolescents 18 years or older provided their own informed consent.

### Data collection

At enrollment the participants provided basic demographic information, completed a questionnaire and additional data was extracted from the medical record. The questionnaire included drug and alcohol use (Youth Risk Behavior Survey) (17), social support (Adolescent Social Support Scale)(18), self-esteem (Rosenberg Self- Esteem Scale)(19), and transition readiness (HIV Adolescent Readiness for Transition Scale (HARTS))(20). The HARTS includes the domains of disclosure, health literacy, health navigation, and self-advocacy based on adolescent’s self-assessment but does not include potential external demographic factors which may also influence transition readiness. The HARTS was developed and validated among adolescents living with perinatally-acquired HIV in South Africa to assist with determining timing and preparation for transition from pediatric to adult care (20). The HARTS was associated with post-transition viral suppression among adolescents, but not among those reporting drug use. Because drug use likely impacts viral suppression more than transition readiness and requires unique care plans, we excluded adolescents who were using drugs from this analysis at the point of determining a transition readiness score. Adolescents were followed until they completed 12 months of care in the adult clinic, at which time we assessed viral suppression (< 200 copies/ml) by review of clinically obtained data.

### Analysis

We first performed bivariable analysis using logistic regression models assessing viral suppression adjusting for potential covariates including: age at ART initiation, ART regimen, biological sex, HIV disclosure status (documentation of the adolescent’s awareness of their own HIV status in the medical record), drug use, alcohol use, social support, self-esteem, and transition readiness (i.e., the HARTS). We then performed a multivariable analysis evaluating viral suppression using multivariable logistic regression with Huber-White robust standard errors, based on the continuous total value of the HARTS and covariates with a p-value of < 0.2 on bivariate analysis. In the multivariable regression, we also assessed for interaction terms among the covariates and retained any interaction terms whose p-value was < 0.05. All statistical analyses were performed using Stata 13 (StataCorp, USA).

### Transition readiness score calculation

We used a point scoring system to develop the final transition readiness score (21). First, we estimated the regression coefficients using the final multivariable model as described above. Next, we organized the individual categorical covariates, HARTS and age at ART initiation, into quartiles and tertiles respectively. We then determined, in regression units, how far each category was from the reference category (β(W-W_REF_)). For each categorical covariate, the lowest category was considered as the reference and the midpoint as the reference value. We then set a base constant (B_constant_) to reflect one point in the point scoring system. For our base constant, we utilized the smallest coefficient (i.e., HARTS) considering the increase in viral suppression associated with a 10-point increase in HARTS (i.e., Beta of 0.05*10). The score for each category of each variable was computed as β(W-W_REF_)/ B_constant_ rounded to the nearest integer. A unit score therefore represents the increase in the likelihood of viral suppression associated with a 10-point increase in the HARTS. Finally, categories were determined according to the range (minimum to maximum) of the total score. Tertiles of this range represent the low-, intermediate-, and high transition readiness levels based on viral suppression one year after transitioning to adult care. We then assessed the performance of the transition readiness tertiles by calculating the sensitivity, specificity, positive and negative predictive values of the high and low transition readiness categories compared to the other two tertiles.

We used a scoring method of converting the regression coefficient for each predictor into an integer. We then multiplied the integer by -1 to easily present transition readiness as predicted probability of viral suppression in practice. To assess model goodness of fit (i.e., agreement between observed and predicted outcomes), we constructed a calibration plot of the two outcomes. Perfect calibration is achieved when points on the graph lie on or around the 45-degree line. We also performed the Homer-Lemeshow Goodness of fit test where p > 0.05 indicates good calibration (22). To evaluate the ability of the model to discriminate between viral suppression and viral failure, we used the concordance statistic (c-index) or the area under the curve (AUC), which represents the probability that a participant with viral suppression is given a higher probability of viral suppression by the model than a randomly chosen subject with viral failure (23). An AUC of 1 indicates perfect discrimination, whereas a value of 0.5 indicates no discriminatory ability. For validation, we re-ran the model using the nonparametric bootstrap method where data was re-sampled 1,000 times with replacement and the bootstrap standard error estimates compared to model estimates (24).

### Ethics statement

 Biomedical Research Ethics Counsel of the University of KwaZulu-Natal, KwaZulu-Natal Department of Health, Partners Healthcare/Massachusetts General Hospital Research Ethics Board and the Emory University Institutional Review Board approved this protocol.

## Results

We prospectively enrolled 199 adolescents with perinatally-acquired HIV during their last visit in the pediatric clinic. Among these participants, the median age at transition was 15 years (range 12–21 years), the median age at ART initiation was 8 years (IQR 5–9 years), and 98 (49%) were female as indicated in Table 1. The majority of participants 144 (72%) were on first-line ART regimens.

**Table 1:** Characteristics of adolescent participants involved in the transition readiness score development

Enrollment characteristics	Adolescent Participants (n = 199)
Median age at enrollment (years) (range)	15 (12–21)
Median age at ART initiation (years) (IQR)	8 (5–9)
Female, n (%)	98 (49%)
First-line ART regimen at transition, n (%)	144 (72%)
Illicit drug use, n (%)	19 (10%)
Documented HIV status disclosure, n (%)	89 (45%)
Alcohol use ever, n (%)	70 (35%)
Median self-esteem score (IQR)	19 (16–22)
Median social-support score (IQR)	39 (30–44)
Median HARTS score (IQR)	31 (21–39)
**Outcomes**	(n = 199)
Viral suppression prior to transition, n (%)	147 (74%)
Viral suppression after transition, n (%)	84 (42%)

Prior to transition 147 (74%) of adolescents had viral suppression, while one year after transition to adult care, 84 (42%) of adolescents had viral suppression. On bivariate analysis, adolescents on first-line ART had higher odds of suppression after transition (OR 10.11; 95% CI 4.06–25.19; p < 0.001) compared to those on second-line ART. Conversely, adolescents who had ever used alcohol (OR 0.34; 95% CI 0.18–0.65; p = 0.001) or were older at ART initiation (OR 0.82; 95% CI 0.73–0.92; p < 0.001) were associated with lower odds of suppression after transition to adult care. On multivariable analysis, being on first-line ART (13.92; 95% CI 4.18–46.40; p < 0.001), having documented HIV status disclosure (AOR 2.75; 95% CI 1.21–6.23; p = 0.015), and higher HARTS (AOR 1.60; 95% CI 1.17–2.21; p = 0.004) were associated with a higher odds of viral suppression after transition, while females (AOR 0.40; 95% CI 0.19–0.85; p = 0.018), those with older age at ART initiation (AOR 0.81; 95% CI 0.71–0.94; p = 0.004), and those who had ever used alcohol (AOR 0.29; 95% CI 0.13–0.68; p = 0.004) were associated with a lower odds of viral suppression after transition as indicated in Table 2.

**Table 2:** Bivariable and multivariable analysis of factors associated with viral suppression one year after transition to adult care for adolescents living with perinatally-acquired HIV

	Bivariable Analysis			Multivariable Analysis		
COVARIATE	OR	95%CI	p-value	AOR	95% CI	p-value
First line ART	**10.11**	**4.06, 25.19**	**< 0.001**	**13.92**	**4.18, 46.40**	< 0.001*
Disclosed HIV status	**1.50**	**0.85, 2.66**	**0.16**	**2.75**	**1.21, 6.23**	0.015*
HARTS score (per unit score)	**1.18**	**0.94, 1.48**	**0.15**	**1.60**†	**1.17, 2.21**	0.004*
Alcohol ever use	**0.34**	**0.18, 0.65**	**0.001**	**0.29**	**0.13, 0.68**	0.004*
Age at ART initiation	**0.82**	**0.73, 0.92**	**< 0.001**	**0.81**	**0.71, 0.94**	0.004*
Drug use	**0.44**	**0.15, 1,29**	**0.13**	**16.26**‡	**0.35, 746.3**	**0.153**
Female	**0.77**	**0.44, 1.36**	**0.37**	**0.40**	**0.19, 0.85**	0.018*
Social support	**1.01**	**0.98, 1.04**	**0.58**	**--**	**--**	**--**
Self-esteem	**1.07**	**0.99, 1.15**	**0.057**	**--**	**--**	**--**

† Coefficient for HARTS score among persons not taking drugs

‡ Coefficient for drug use among persons scoring 0 on HARTS score

*p-value <0.05

## Transition readiness score development

Using the risk factors associated with viral suppression after transition to adult care in the multivariable model including age at ART initiation, ART regimen, HIV disclosure, HARTS, Alcohol use, and biological sex (Table 2), we created a transition readiness score as indicated by Table 3.

**Table 3:** Transition readiness score calculation for viral suppression one year after transitioning to adult care for adolescents living with perinatally-acquired HIV

Variable	Beta	Categories	Reference value (W)	β(W-W_REF_)	Points = (β(W-W_REF_) /B_constant_)* ^−^1
HARTS	0.05	2–20*	11	0	0
		21–30	25.5	0.73	1
		31–39	35	1.20	2
		40–56	48	1.85	4
Age at ART initiation	-0.21	0–5*	2.5	0	0
		6–8	7	-0.95	-2
		9–15	12	-2.0	-4
Regimen line	2.63	Second line	0 = ref	0	0
		First line	1	2.63	5
Sex	-0.91	Male	0 = ref	0	0
		Female	1	-0.91	-2
Disclosed	1.01	No	0 = ref	0	0
		Yes	1	1.01	2
Alcohol use	-1.23	No	0 = ref	0	0
		Yes	1	-1.23	-2
Total score range					-8 to 11

*Reference category

For HARTS B_constant_ we considered a 10-point increase in HARTs score (0.05*10 = 0.5)

Model (log scale) = 0.05*HARTS Score − 0.21* Age at ART initiation + 2.63*first line ART − 0.91* Female + 1.01*Documented Disclosure − 1.23*alcohol use − 1.92

**Fig. 1:**
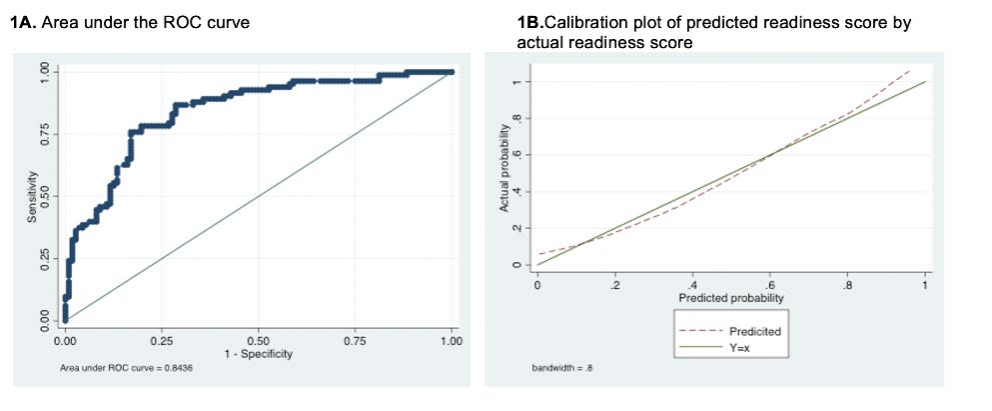
A. Area under the curve of the Receiver Operating Characteristic (ROC) curve and B. calibration plot of predicted readiness scores by actual readiness score 1 A. Area under the ROC curve 1B.Calibration plot of predicted readiness score by actual readiness score

For the final model, the Hosmer-Lemeshow p value was 0.36 and the calibration plot fell along the 45-degree line suggesting good agreement between the predicted and actual probability of viral suppression as indicated by Fig. 1. Comparing the standard and bootstrap models, there was no significant difference in standard errors for all variables, except the interaction term which lost statistical significance. For the overall model, the area under the curve was 0.84 (95% CI 0.79–0.90) indicating moderate discrimination (Fig. 1).

### Transition readiness score use

The transition readiness score ranges from − 8 to 11. Participants scoring ≥ 5 are considered to have high transition readiness with the highest odds for viral suppression one year after transition to adult care. Those scoring between − 1 and 4 are at intermediate transition readiness, while those scoring ≤-2 have low transition readiness and have the lowest odds for viral suppression after transition. The overall sensitivity of the intermediate/high transition readiness category (>-2) compared to low transition readiness category (≤-2) in determining viral suppression one year after transitioning to adult care was 96.4% (95% CI 89.9–99.3) with a specificity of 27.7% (95% CI 19.6–36.9) and a negative predictive value of 91.2% (95% CI 76.3–98.1) positive predictive value of 50.0% (95%CI 42.1–57.9) as indicated in Table 4. For the high transition readiness category (≥ 5) compared to intermediate and low transition readiness (< 5), the transition readiness score had a sensitivity of 56.0 (95% CI 44.7–66.8), specificity of 86.6 (95% CI 78.9–92.3), positive predictive value of 75.8 (95% CI 63.3–85.8) and a negative predictive value or 72.4% (95% CI 64.0–79.8) for the prediction of viral suppression one year after transition to adult care.

**Table 4:** Sensitivity, specificity, positive and negative predictive value of the transition readiness tertiles

Transition readiness	Sensitivity (95% CI)	Specificity (95% CI)	Positive predictive value (95% CI)	Negative predictive value (95% CI)
High (≥ 5) vs. intermediate and low (< 5)	56.0 (44.7–66.8)	86.6 (78.9–92.3)	75.8 (63.3–85.8)	72.4 (64.0–79.8)
Intermediate and high (>-2) vs. low (≤-2)	96.4 (89.9–99.3)	27.7 (19.6–36.9)	50.0 (42.1–57.9)	91.2 (76.3–98.1)

## Discussion

We present the development of a transition readiness score through a prospective analysis of factors associated with viral suppression among adolescents living with perinatally-acquired HIV transitioning to adult care. To our knowledge, this is the first transition readiness score assessment for this population. This clinical prediction tool can be useful to clinicians by providing evidence-based decision support around timing of transition to adult care for adolescents living with perinatally-acquired HIV in South Africa and potentially in other similar settings. By creating high, intermediate, and low transition readiness tertiles, clinicians through the administration of a brief questionnaire evaluating drug use and transition readiness (HARTS) combined with demographic information (age at ART initiation, biological sex, ART regimen, and HIV disclosure status), can determine which adolescents are likely ready for transition to adult care and which adolescents may need additional services prior to transition.

Based on the sensitivity and specificity analysis, we suggest using the transition readiness score in addition to clinical judgement to determine timing of transition from pediatric to adult care. We favor the high specificity of scoring ≥ 5 and its significant association with viral suppression after transition as the binary threshold. Therefore, adolescents scoring in the high range (≥ 5) are likely ready to transition to adult care without additional intervention with a high likelihood of viral suppression one year after transition. Adolescents scoring in the intermediate and low range (< 5) would likely benefit from additional time in pediatric care or targeted interventions to address modifiable areas of deficiency. We plan to use this assessment in future studies to provide differentiated care for adolescents at higher risk of failure during transitioning to adult care.

Currently, there are no clear guidelines on when to transition adolescents and how to best determine transition readiness for adolescents living with HIV in South Africa (20). In most settings, age is used as the determining factor for the timing of transition to adult care. However, our analysis found that age at the time of transition was not associated with viral suppression after transition. We did find that older age at the time of ART initiation was significantly associated viral failure after transition; however, this factor is not modifiable at the time of transition. Benefits of early ART initiation among children with perinatally-acquired HIV seem to extend beyond clinical, immunological, and viral outcomes and continue to improve psychosocial development into adolescence (25).

We found that the multidomain transition readiness assessment (i.e., the HARTS) carried significant weight in predicting viral suppression after transition to adult care (20). Other transition readiness assessments have been developed and used for pediatric chronic illnesses in North America; however, these were not designed with the specific needs and environment of adolescents living with perinatally-acquired HIV (14–16). The HARTS was developed and validated specifically for adolescents living with perinatally-acquired HIV in sub-Saharan Africa, making it a clinically relevant assessment tool for adolescent transition in this population (20). The HARTS contains four modifiable domains that have been associated with viral suppression after transition to adult care: disclosure, health navigation, health literacy, and self-advocacy. These are all areas that could be strengthened among adolescents in preparation for transition to adult care.

We found that disclosure, the events leading an adolescent to becoming aware of their own HIV status, was important to transition readiness. In this study, disclosure was evaluated by two separate methods. Disclosure was first evaluated as one of the HARTS domains to evaluate self-assessed knowledge of disclosure and second by documentation in the medical record to evaluate clinical team’s assessment of and involvement in the adolescent’s understanding of their diagnosis. Without knowledge of their HIV status, adolescents cannot participate in peer support activities that can adversely affect self-esteem and mental health resulting in decreased engagement in care. Disclosure has direct effects on internalized stigma, mental health and other factors that can assist adolescents during transition to adult care (26, 27). Documented benefits of early, successful disclosure have been shown to improve ART adherence, engagement in care, and immunologic response, while contributing to lower mortality compared to adolescents who were not disclosed of their HIV status (28–30).

Without additional intervention, adolescents who have already failed first-line ART treatment continue to have high rates of failure likely due to ongoing poor adherence or ART resistance (31, 32). Although not modifiable in the transition readiness score, adolescents who are already on second-line ART at the time of transition would likely benefit from adherence interventions prior to transition given their higher risk of viral failure one year after transitioning to adult care. Similarly, we found that females had a higher risk of viral failure after transition to adult care. This gender disparity in viral suppression has been seen in pediatric and adolescent cohorts in South Africa (33, 34). Although non-modifiable for the transition readiness score, gender-based interventions prior to transition may assist females during the transition process.

This transition readiness score is designed for adolescents who are not using drugs. Drug use has been associated with poor adherence and viral failure among adolescents in other studies (35, 36). Although not significant in our study, likely due to small numbers of adolescents actively reporting drug use, we feel drug use is an important factor in assessing transition readiness. For adolescents using drugs, efforts should focus on addiction services, drug counseling, and medication adherence prior to transitioning these adolescents to adult care. Adolescents who have ever used alcohol, although not directly modifiable, would likely also benefit from counseling prior to transition to adult care.

This study has several limitations. The study was conducted in a single population with a younger age range (median of 15 years old) for transition to adult care. Future studies will be needed to assess transition risk among adolescents who transition at older ages, in settings outside South Africa, and among adolescents with non-perinatally-acquired HIV. We also used convenience sampling to enroll participants, although most eligible participants agreed to participate; this approach could have introduced bias into the sample. However, baseline demographics were similar to other clinics in the region (11, 20). In addition, we evaluated viral suppression one year after transition to adult care. Several studies have found that retention in care and viral suppression rates decline over time after transition to adult care; therefore, longer follow-up time may be required. However, our readiness score can be helpful to identify those adolescents who may be at higher risk for early poor outcomes after transition and may need additional services prior to transition. This score is not intended to replace clinical judgement and it does have limitations in predicting successful transition to adult care. Additional studies will be needed to further validate and refine the transition risk score, as well as, to identify factors associated with viral failure despite high transition readiness scores.

## Conclusions

We developed a transition readiness score for adolescents living with perinatally-acquired HIV to identify which adolescents are ready to transition to adult care and who may need additional resources prior to transition. Modifiable factors such as transition readiness, disclosure, and drug/alcohol use can be optimized prior to transition to help improve one-year viral suppression outcomes among adolescents after transition to adult care.

## Data Availability

Data and analysis coding will be made available by request.
